# Dicarbon­yl[2-hydr­oxy-3,5,7-tris­(mor­pho­linomethyl)cyclo­hepta-2,4,6-trien­onato(1–)-κ^2^O^1^,O^2^]rhodium(I)

**DOI:** 10.1107/S160053680803780X

**Published:** 2008-11-20

**Authors:** Tania N. Hill, G. Steyl

**Affiliations:** a Department of Chemistry, University of the Free State, Bloemfontein 9300, South Africa

## Abstract

In the title compound, [Rh(C_22_H_32_N_3_O_5_)(CO)_2_], the Rh^I^ atom is coordinated by two carbonyl ligands and two tropolonate O atoms in a distorted square-planar geometry. It is an example of a new type of tropolone derivative that has not been characterized *via* solid-state methods. Weak intra­molecular C—H⋯N and inter­molecular C—H⋯O hydrogen bonds, and π–π stacking inter­actions between the tropolone rings [centroid–centroid distance = 3.590 (8) Å] are observed in the crystal structure.

## Related literature

For general background, see: Banwell *et al.* (1992 ▶); Boguszewska-Chachulska *et al.* (2006 ▶); Burgstein *et al.* (1998 ▶); Crous *et al.* (2005 ▶); Dewar (1945 ▶); Kierst *et al.* (1982 ▶). For a related structure, see: Steyl *et al.* (2004 ▶).
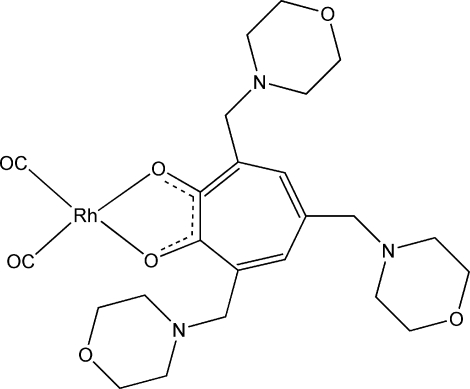


## Experimental

### 

#### Crystal data

[Rh(C_22_H_32_N_3_O_5_)(CO)_2_]
*M* *_r_* = 577.44
Monoclinic, 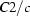

*a* = 17.7889 (6) Å
*b* = 16.6106 (5) Å
*c* = 17.7279 (4) Å
β = 105.772 (1)°
*V* = 5041.1 (3) Å^3^
*Z* = 8
Mo *K*α radiation
μ = 0.73 mm^−1^
*T* = 100 (2) K
0.15 × 0.06 × 0.05 mm

#### Data collection

Bruker X8 APEXII Kappa CCD diffractometer
Absorption correction: multi-scan (*SADABS*; Bruker, 2001 ▶) *T*_min_ = 0.901, *T*_max_ = 0.966
36747 measured reflections
5450 independent reflections
4616 reflections with *I* > 2σ(*I*)
*R*_int_ = 0.06

#### Refinement

*R*[*F* ^2^ > 2σ(*F* ^2^)] = 0.034
*wR*(*F* ^2^) = 0.070
*S* = 1.01
5450 reflections
316 parameters
H-atom parameters constrained
Δρ_max_ = 0.58 e Å^−3^
Δρ_min_ = −0.55 e Å^−3^

### 

Data collection: *APEX2* (Bruker, 2007 ▶); cell refinement: *SAINT-Plus* (Bruker, 2007 ▶); data reduction: *SAINT-Plus*; program(s) used to solve structure: *SHELXS97* (Sheldrick, 2008 ▶); program(s) used to refine structure: *SHELXL97* (Sheldrick, 2008 ▶); molecular graphics: *DIAMOND* (Brandenburg & Putz, 1999 ▶); software used to prepare material for publication: *WinGX* (Farrugia, 1999 ▶).

## Supplementary Material

Crystal structure: contains datablocks global, I. DOI: 10.1107/S160053680803780X/hy2163sup1.cif

Structure factors: contains datablocks I. DOI: 10.1107/S160053680803780X/hy2163Isup2.hkl

Additional supplementary materials:  crystallographic information; 3D view; checkCIF report

## Figures and Tables

**Table 1:** Selected bond lengths (Å)

Rh1—C01	1.835 (3)
Rh1—C02	1.840 (2)
Rh1—O1	2.0209 (16)
Rh1—O2	2.0212 (15)

**Table 2:** Hydrogen-bond geometry (Å, °)

*D*—H⋯*A*	*D*—H	H⋯*A*	*D*⋯*A*	*D*—H⋯*A*
C4—H4⋯N32	0.95	2.28	2.760 (3)	110
C6—H6⋯N72	0.95	2.31	2.785 (3)	110
C36—H36*A*⋯O01^i^	0.99	2.59	3.497 (3)	153
C53—H53*A*⋯O55^ii^	0.99	2.56	3.509 (4)	161

Symmetry codes: (i) 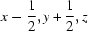; (ii) 
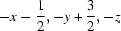
.

**Fig. 1.**
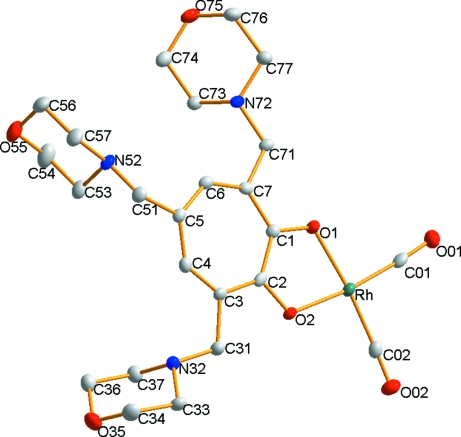
Molecular structure of the title compound. Displacement ellipsoids are drawn at the 50% probability level. H atoms have been omitted for clarity.

## supplementary crystallographic information

### Comment

Tropolone type compounds have been of interest since its first discovery in the early 1940's (Dewar, 1945), with applications in pharmacology (Banwell *et al.*,  1992; Kierst *et al.*,  1982) and catalysis (Burgstein *et al.*,  1998; Crous *et al.*,  2005). A recent report on the anti-viral activity of morpholine derivatives of tropolone (Doering Knox) indicated moderate to strong activity against the hepatitis C virus strain (Boguszewska-Chachulska *et al.*, 2006). The addition of morpholine groups to a compound increases its water solubilty properties and thus simplifying the method of dosage, *i.e.*, pallative. Although this compound has been extensively studied, the preferred orientation of the morpholine groups are unknown, as well as the geometrical properties of the tropolone ring system. In this regard, we present a dicarbonyl rhodium(I) complex of a 3,5,7-tris(methylmorpholine)tropolonate ligand (Fig. 1; Table 1).

The molecular packing of the title compound is strongly influenced by the morpholine moieties as these form extensive hydrogen bonding networks (Table 2). A close Rh1···Rh1^i^ contact [3.2826 (3)Å; symmetry code: (i) 1-x, y, 0.5-z] exists between associated metal centres. This short contact is stabilized by π–π stacking between the corresponding cycloheptatriene rings, with a centroid–centroid distance of 3.590 (8)Å and an interplanar angle of 3.99 (5)°. The slight twist of the two cycloheptatriene ring systems can be attributed to the methylmorpholine functional groups creating a sterically crowded environment.

The crystal packing of diketonate dicarbonyl rhodium(I) complexes tends to favour a head-to-tail packing mode. The [Rh(tropolonate)(CO)_2_] complex (Steyl *et al.*, 2004) was deemed to be a singular occurance of the head-to-head packing mode of these molecular systems. The title compound exhibits a slightly distorted orientation as defined by the O1—Rh1—Rh1^i^—O2^i^ torsion angle of 37.09 (3)°. This observation is surprising since the addition of bulky groups on the 3,7-positions was expected to force the molecular system in the head-to-tail packing mode. The π–π stacking and hydrogen bonding interactions stabilize the crystal structure.

### Experimental

The title compound was synthesized by the addition of 3,5,7-tris(methylmorpholine)tropolone (0.083 g, 0.32 mmol) to an acetone solution of [Rh(µ-Cl)(CO)_2_]_2_ (0.100 g, 0.29 mmol). On slow evaporation of the solvent, crystals suitable for X-ray analysis was obtained (yield 30%, 0.045 g).

### Refinement

H atoms were positioned geometrically and refined as riding atoms, with C—H = 0.95 (CH) and 0.99 (CH_2_) Å and *U*_iso_(H) = 1.2*U*_eq_(C).

### Crystal data

**Table:**

[Rh(C_22_H_32_N_3_O_5_)(CO)_2_]	*F*_000_ = 2384
*M**_r_* = 577.44	*D*_x_ = 1.522 Mg m^−^^3^
Monoclinic, *C*2/*c*	Mo *K*α radiation λ = 0.71073 Å
Hall symbol: -C 2yc	Cell parameters from 7078 reflections
*a* = 17.7889 (6) Å	θ = 2.4–26.3º
*b* = 16.6106 (5) Å	µ = 0.73 mm^−^^1^
*c* = 17.7279 (4) Å	*T* = 100 (2) K
β = 105.772 (1)º	Needle, yellow
*V* = 5041.1 (3) Å^3^	0.15 × 0.06 × 0.05 mm
*Z* = 8	

### Data collection

**Table:**

Bruker X8 APEXII Kappa CCD diffractometer	4616 reflections with *I* > 2σ(*I*)
Radiation source: sealed tube	*R*_int_ = 0.06
Monochromator: graphite	θ_max_ = 27.0º
ω and φ scans	θ_min_ = 1.9º
Absorption correction: multi-scan(SADABS; Bruker, 2001)	*h* = −23→22
*T*_min_ = 0.901, *T*_max_ = 0.966	*k* = −22→22
36747 measured reflections	*l* = −23→21
5450 independent reflections	

### Refinement

**Table:**

Refinement on *F*^2^	H-atom parameters constrained
Least-squares matrix: full	*w* = 1/[σ^2^(*F*_o_^2^) + (0.0228*P*)^2^ + 6.7535*P*] where *P* = (*F*_o_^2^ + 2*F*_c_^2^)/3
*R*[*F*^2^ > 2σ(*F*^2^)] = 0.034	(Δ/σ)_max_ = 0.001
*wR*(*F*^2^) = 0.070	Δρ_max_ = 0.58 e Å^−^^3^
*S* = 1.01	Δρ_min_ = −0.55 e Å^−^^3^
5450 reflections	Extinction correction: none
316 parameters	

### Fractional atomic coordinates and isotropic or equivalent isotropic displacement parameters (Å^2^)

**Table:**

	*x*	*y*	*z*	*U*_iso_*/*U*_eq_	
Rh1	0.005201 (12)	0.212518 (10)	0.158973 (11)	0.01523 (6)	
N32	−0.29013 (12)	0.40449 (12)	0.14152 (12)	0.0184 (4)	
N52	−0.09972 (13)	0.68641 (11)	0.11782 (12)	0.0193 (5)	
N72	0.12652 (12)	0.55064 (11)	0.12939 (11)	0.0161 (4)	
O1	−0.09251 (10)	0.27724 (9)	0.15271 (9)	0.0164 (4)	
O2	0.04571 (10)	0.32572 (9)	0.15522 (9)	0.0168 (4)	
O35	−0.44856 (11)	0.45215 (11)	0.09597 (11)	0.0291 (4)	
O55	−0.14421 (12)	0.81072 (10)	0.00353 (12)	0.0338 (5)	
O75	0.19657 (11)	0.67733 (10)	0.06510 (10)	0.0272 (4)	
C01	0.10082 (16)	0.16569 (13)	0.16882 (14)	0.0186 (5)	
C02	−0.04121 (15)	0.11321 (14)	0.15654 (14)	0.0191 (5)	
O01	0.16289 (11)	0.14149 (10)	0.17892 (10)	0.0266 (4)	
O02	−0.07249 (11)	0.05334 (10)	0.15587 (11)	0.0271 (4)	
C1	−0.00561 (14)	0.38233 (13)	0.15364 (13)	0.0139 (5)	
C2	−0.08253 (15)	0.35538 (13)	0.15345 (13)	0.0153 (5)	
C3	−0.14821 (14)	0.40295 (13)	0.15290 (13)	0.0148 (5)	
C4	−0.15152 (15)	0.48618 (13)	0.15770 (13)	0.0164 (5)	
H4	−0.2013	0.5066	0.158	0.02*	
C5	−0.09482 (14)	0.54493 (13)	0.16221 (13)	0.0153 (5)	
C6	−0.01989 (14)	0.53233 (14)	0.15582 (13)	0.0154 (5)	
H6	0.0094	0.5803	0.1565	0.018*	
C7	0.02014 (14)	0.46255 (13)	0.14867 (13)	0.0144 (5)	
C31	−0.22148 (14)	0.35437 (14)	0.15082 (15)	0.0201 (5)	
H31A	−0.231	0.3155	0.1069	0.024*	
H31B	−0.2127	0.3232	0.2001	0.024*	
C33	−0.34489 (15)	0.37470 (14)	0.18290 (15)	0.0212 (6)	
H33A	−0.3175	0.3657	0.2388	0.025*	
H33B	−0.3675	0.3228	0.16	0.025*	
C34	−0.40884 (16)	0.43614 (16)	0.17571 (16)	0.0263 (6)	
H34A	−0.4468	0.4161	0.2031	0.032*	
H34B	−0.386	0.4868	0.2015	0.032*	
C36	−0.39560 (15)	0.47768 (15)	0.05269 (15)	0.0236 (6)	
H36A	−0.3727	0.5303	0.073	0.028*	
H36B	−0.4245	0.4848	−0.0031	0.028*	
C37	−0.33140 (15)	0.41744 (15)	0.05905 (14)	0.0222 (6)	
H37A	−0.3537	0.3659	0.035	0.027*	
H37B	−0.2944	0.4374	0.0305	0.027*	
C51	−0.11655 (15)	0.63133 (13)	0.17494 (14)	0.0184 (5)	
H51A	−0.173	0.6339	0.1716	0.022*	
H51B	−0.0873	0.6486	0.2283	0.022*	
C53	−0.15194 (17)	0.67127 (14)	0.04011 (14)	0.0239 (6)	
H53A	−0.2069	0.6774	0.0416	0.029*	
H53B	−0.1447	0.6155	0.0238	0.029*	
C54	−0.1347 (2)	0.72976 (16)	−0.01803 (17)	0.0348 (7)	
H54A	−0.0804	0.7217	−0.021	0.042*	
H54B	−0.1703	0.719	−0.0706	0.042*	
C56	−0.09308 (18)	0.82638 (16)	0.07909 (17)	0.0344 (7)	
H56A	−0.1001	0.8827	0.0943	0.041*	
H56B	−0.0383	0.8198	0.0773	0.041*	
C57	−0.10926 (17)	0.76990 (14)	0.13928 (16)	0.0267 (6)	
H57A	−0.0729	0.7815	0.1912	0.032*	
H57B	−0.1632	0.7783	0.143	0.032*	
C71	0.10184 (14)	0.46875 (13)	0.13924 (14)	0.0159 (5)	
H71A	0.1388	0.4449	0.186	0.019*	
H71B	0.1047	0.4365	0.0931	0.019*	
C73	0.09197 (15)	0.58095 (14)	0.04982 (14)	0.0186 (5)	
H73A	0.0344	0.5756	0.0361	0.022*	
H73B	0.1115	0.5493	0.0117	0.022*	
C74	0.11413 (16)	0.66843 (14)	0.04648 (15)	0.0226 (6)	
H74A	0.0902	0.6898	−0.0068	0.027*	
H74B	0.0937	0.6999	0.084	0.027*	
C76	0.23202 (16)	0.64617 (15)	0.14098 (15)	0.0254 (6)	
H76A	0.2148	0.6782	0.1804	0.031*	
H76B	0.2895	0.6514	0.1524	0.031*	
C77	0.21100 (15)	0.55855 (14)	0.14762 (15)	0.0214 (6)	
H77A	0.231	0.5256	0.1107	0.026*	
H77B	0.2353	0.5389	0.2015	0.026*	

### Atomic displacement parameters (Å^2^)

**Table:**

	*U*^11^	*U*^22^	*U*^33^	*U*^12^	*U*^13^	*U*^23^
Rh1	0.01874 (11)	0.01015 (9)	0.01714 (10)	0.00082 (8)	0.00544 (7)	0.00046 (8)
N32	0.0151 (12)	0.0180 (10)	0.0232 (11)	0.0017 (8)	0.0071 (9)	0.0042 (8)
N52	0.0243 (13)	0.0108 (9)	0.0241 (12)	0.0005 (8)	0.0087 (9)	0.0007 (8)
N72	0.0160 (12)	0.0147 (10)	0.0173 (11)	−0.0031 (8)	0.0044 (9)	0.0002 (8)
O1	0.0176 (9)	0.0104 (8)	0.0215 (9)	−0.0011 (7)	0.0059 (7)	0.0009 (6)
O2	0.0169 (10)	0.0116 (8)	0.0234 (9)	0.0009 (7)	0.0082 (7)	0.0007 (7)
O35	0.0178 (11)	0.0348 (11)	0.0350 (11)	0.0033 (8)	0.0075 (9)	0.0029 (8)
O55	0.0412 (14)	0.0205 (10)	0.0409 (12)	0.0053 (9)	0.0134 (10)	0.0108 (8)
O75	0.0316 (12)	0.0229 (9)	0.0280 (11)	−0.0096 (8)	0.0094 (9)	0.0049 (8)
C01	0.0276 (16)	0.0111 (11)	0.0180 (13)	−0.0013 (11)	0.0075 (11)	−0.0012 (9)
C02	0.0227 (15)	0.0181 (13)	0.0178 (13)	0.0044 (11)	0.0074 (11)	0.0012 (10)
O01	0.0241 (12)	0.0217 (9)	0.0331 (11)	0.0048 (8)	0.0065 (9)	0.0003 (8)
O02	0.0307 (12)	0.0167 (9)	0.0368 (11)	−0.0040 (8)	0.0141 (9)	−0.0027 (8)
C1	0.0171 (14)	0.0143 (11)	0.0112 (11)	−0.0002 (10)	0.0055 (10)	−0.0017 (9)
C2	0.0229 (14)	0.0124 (11)	0.0102 (11)	−0.0016 (10)	0.0036 (10)	0.0013 (9)
C3	0.0163 (13)	0.0152 (11)	0.0136 (12)	−0.0015 (10)	0.0053 (10)	0.0029 (9)
C4	0.0174 (14)	0.0169 (12)	0.0150 (12)	0.0032 (10)	0.0047 (10)	0.0014 (9)
C5	0.0191 (14)	0.0134 (11)	0.0134 (12)	0.0003 (10)	0.0042 (10)	0.0002 (9)
C6	0.0195 (14)	0.0147 (11)	0.0124 (12)	−0.0032 (10)	0.0052 (10)	−0.0004 (9)
C7	0.0150 (14)	0.0167 (11)	0.0112 (12)	−0.0031 (9)	0.0028 (9)	−0.0020 (9)
C31	0.0186 (14)	0.0156 (12)	0.0266 (14)	−0.0001 (10)	0.0069 (11)	0.0029 (10)
C33	0.0209 (15)	0.0174 (12)	0.0274 (14)	−0.0023 (10)	0.0102 (11)	0.0027 (10)
C34	0.0248 (16)	0.0270 (14)	0.0319 (16)	0.0018 (12)	0.0160 (13)	0.0032 (11)
C36	0.0230 (16)	0.0245 (14)	0.0227 (14)	0.0035 (11)	0.0051 (11)	0.0013 (11)
C37	0.0216 (15)	0.0230 (13)	0.0224 (14)	0.0026 (11)	0.0068 (11)	−0.0003 (10)
C51	0.0223 (15)	0.0149 (11)	0.0196 (13)	0.0022 (10)	0.0083 (11)	−0.0022 (10)
C53	0.0314 (17)	0.0162 (12)	0.0242 (14)	0.0038 (11)	0.0075 (12)	0.0005 (10)
C54	0.049 (2)	0.0275 (15)	0.0313 (16)	0.0108 (13)	0.0167 (14)	0.0063 (12)
C56	0.0318 (18)	0.0175 (13)	0.052 (2)	−0.0038 (12)	0.0075 (15)	0.0067 (12)
C57	0.0326 (17)	0.0140 (12)	0.0319 (15)	0.0007 (11)	0.0060 (12)	−0.0026 (10)
C71	0.0185 (14)	0.0131 (11)	0.0163 (12)	−0.0027 (10)	0.0054 (10)	−0.0018 (9)
C73	0.0207 (15)	0.0176 (12)	0.0172 (13)	−0.0026 (10)	0.0045 (10)	−0.0004 (9)
C74	0.0294 (17)	0.0188 (13)	0.0188 (13)	−0.0043 (11)	0.0050 (11)	0.0003 (10)
C76	0.0214 (15)	0.0232 (13)	0.0309 (15)	−0.0067 (11)	0.0057 (12)	0.0027 (11)
C77	0.0193 (15)	0.0186 (12)	0.0261 (14)	−0.0026 (10)	0.0060 (11)	0.0000 (10)

### Geometric parameters (Å, °)

**Table:**

Rh1—C01	1.835 (3)	C31—H31B	0.99
Rh1—C02	1.840 (2)	C33—C34	1.508 (3)
Rh1—O1	2.0209 (16)	C33—H33A	0.99
Rh1—O2	2.0212 (15)	C33—H33B	0.99
N32—C31	1.450 (3)	C34—H34A	0.99
N32—C33	1.456 (3)	C34—H34B	0.99
N32—C37	1.463 (3)	C36—C37	1.499 (3)
N52—C51	1.455 (3)	C36—H36A	0.99
N52—C53	1.459 (3)	C36—H36B	0.99
N52—C57	1.460 (3)	C37—H37A	0.99
N72—C71	1.454 (3)	C37—H37B	0.99
N72—C77	1.455 (3)	C51—H51A	0.99
N72—C73	1.466 (3)	C51—H51B	0.99
O1—C2	1.310 (3)	C53—C54	1.507 (4)
O2—C1	1.306 (3)	C53—H53A	0.99
O35—C34	1.423 (3)	C53—H53B	0.99
O35—C36	1.431 (3)	C54—H54A	0.99
O55—C54	1.421 (3)	C54—H54B	0.99
O55—C56	1.424 (3)	C56—C57	1.506 (4)
O75—C76	1.419 (3)	C56—H56A	0.99
O75—C74	1.421 (3)	C56—H56B	0.99
C01—O01	1.143 (3)	C57—H57A	0.99
C02—O02	1.138 (3)	C57—H57B	0.99
C1—C7	1.419 (3)	C71—H71A	0.99
C1—C2	1.439 (3)	C71—H71B	0.99
C2—C3	1.408 (3)	C73—C74	1.511 (3)
C3—C4	1.387 (3)	C73—H73A	0.99
C3—C31	1.525 (3)	C73—H73B	0.99
C4—C5	1.390 (3)	C74—H74A	0.99
C4—H4	0.95	C74—H74B	0.99
C5—C6	1.384 (3)	C76—C77	1.515 (3)
C5—C51	1.519 (3)	C76—H76A	0.99
C6—C7	1.384 (3)	C76—H76B	0.99
C6—H6	0.95	C77—H77A	0.99
C7—C71	1.511 (3)	C77—H77B	0.99
C31—H31A	0.99		
			
C01—Rh1—C02	91.18 (11)	N32—C37—C36	109.8 (2)
C01—Rh1—O1	172.67 (8)	N32—C37—H37A	109.7
C02—Rh1—O1	95.85 (9)	C36—C37—H37A	109.7
C01—Rh1—O2	93.89 (9)	N32—C37—H37B	109.7
C02—Rh1—O2	174.27 (9)	C36—C37—H37B	109.7
O1—Rh1—O2	79.18 (6)	H37A—C37—H37B	108.2
C31—N32—C33	113.94 (19)	N52—C51—C5	112.45 (19)
C31—N32—C37	112.11 (19)	N52—C51—H51A	109.1
C33—N32—C37	109.3 (2)	C5—C51—H51A	109.1
C51—N52—C53	110.49 (19)	N52—C51—H51B	109.1
C51—N52—C57	110.82 (19)	C5—C51—H51B	109.1
C53—N52—C57	108.7 (2)	H51A—C51—H51B	107.8
C71—N72—C77	112.65 (19)	N52—C53—C54	109.8 (2)
C71—N72—C73	112.05 (18)	N52—C53—H53A	109.7
C77—N72—C73	108.68 (19)	C54—C53—H53A	109.7
C2—O1—Rh1	114.49 (15)	N52—C53—H53B	109.7
C1—O2—Rh1	114.66 (14)	C54—C53—H53B	109.7
C34—O35—C36	111.5 (2)	H53A—C53—H53B	108.2
C54—O55—C56	109.5 (2)	O55—C54—C53	111.4 (2)
C76—O75—C74	110.30 (19)	O55—C54—H54A	109.3
O01—C01—Rh1	174.5 (2)	C53—C54—H54A	109.3
O02—C02—Rh1	177.1 (2)	O55—C54—H54B	109.3
O2—C1—C7	116.2 (2)	C53—C54—H54B	109.3
O2—C1—C2	115.77 (19)	H54A—C54—H54B	108
C7—C1—C2	127.9 (2)	O55—C56—C57	110.9 (2)
O1—C2—C3	116.5 (2)	O55—C56—H56A	109.5
O1—C2—C1	115.8 (2)	C57—C56—H56A	109.5
C3—C2—C1	127.7 (2)	O55—C56—H56B	109.5
C4—C3—C2	127.4 (2)	C57—C56—H56B	109.5
C4—C3—C31	118.6 (2)	H56A—C56—H56B	108
C2—C3—C31	113.90 (19)	N52—C57—C56	110.4 (2)
C3—C4—C5	131.4 (2)	N52—C57—H57A	109.6
C3—C4—H4	114.3	C56—C57—H57A	109.6
C5—C4—H4	114.3	N52—C57—H57B	109.6
C6—C5—C4	126.1 (2)	C56—C57—H57B	109.6
C6—C5—C51	116.4 (2)	H57A—C57—H57B	108.1
C4—C5—C51	117.5 (2)	N72—C71—C7	114.07 (19)
C7—C6—C5	131.7 (2)	N72—C71—H71A	108.7
C7—C6—H6	114.2	C7—C71—H71A	108.7
C5—C6—H6	114.2	N72—C71—H71B	108.7
C6—C7—C1	126.7 (2)	C7—C71—H71B	108.7
C6—C7—C71	119.2 (2)	H71A—C71—H71B	107.6
C1—C7—C71	114.0 (2)	N72—C73—C74	108.80 (19)
N32—C31—C3	112.75 (19)	N72—C73—H73A	109.9
N32—C31—H31A	109	C74—C73—H73A	109.9
C3—C31—H31A	109	N72—C73—H73B	109.9
N32—C31—H31B	109	C74—C73—H73B	109.9
C3—C31—H31B	109	H73A—C73—H73B	108.3
H31A—C31—H31B	107.8	O75—C74—C73	110.8 (2)
N32—C33—C34	108.66 (19)	O75—C74—H74A	109.5
N32—C33—H33A	110	C73—C74—H74A	109.5
C34—C33—H33A	110	O75—C74—H74B	109.5
N32—C33—H33B	110	C73—C74—H74B	109.5
C34—C33—H33B	110	H74A—C74—H74B	108.1
H33A—C33—H33B	108.3	O75—C76—C77	111.6 (2)
O35—C34—C33	111.7 (2)	O75—C76—H76A	109.3
O35—C34—H34A	109.3	C77—C76—H76A	109.3
C33—C34—H34A	109.3	O75—C76—H76B	109.3
O35—C34—H34B	109.3	C77—C76—H76B	109.3
C33—C34—H34B	109.3	H76A—C76—H76B	108
H34A—C34—H34B	107.9	N72—C77—C76	109.4 (2)
O35—C36—C37	111.1 (2)	N72—C77—H77A	109.8
O35—C36—H36A	109.4	C76—C77—H77A	109.8
C37—C36—H36A	109.4	N72—C77—H77B	109.8
O35—C36—H36B	109.4	C76—C77—H77B	109.8
C37—C36—H36B	109.4	H77A—C77—H77B	108.3
H36A—C36—H36B	108		
			
C02—Rh1—O1—C2	179.63 (15)	C31—N32—C33—C34	174.1 (2)
O2—Rh1—O1—C2	−3.26 (14)	C37—N32—C33—C34	−59.6 (3)
C01—Rh1—O2—C1	−175.31 (16)	C36—O35—C34—C33	−56.2 (3)
O1—Rh1—O2—C1	2.28 (15)	N32—C33—C34—O35	58.3 (3)
Rh1—O2—C1—C7	−178.22 (15)	C34—O35—C36—C37	55.3 (3)
Rh1—O2—C1—C2	−1.0 (2)	C31—N32—C37—C36	−172.9 (2)
Rh1—O1—C2—C3	−177.12 (15)	C33—N32—C37—C36	59.7 (3)
Rh1—O1—C2—C1	3.7 (2)	O35—C36—C37—N32	−57.1 (3)
O2—C1—C2—O1	−1.8 (3)	C53—N52—C51—C5	68.7 (3)
C7—C1—C2—O1	175.0 (2)	C57—N52—C51—C5	−170.8 (2)
O2—C1—C2—C3	179.1 (2)	C6—C5—C51—N52	51.4 (3)
C7—C1—C2—C3	−4.1 (4)	C4—C5—C51—N52	−128.3 (2)
O1—C2—C3—C4	176.3 (2)	C51—N52—C53—C54	179.0 (2)
C1—C2—C3—C4	−4.7 (4)	C57—N52—C53—C54	57.2 (3)
O1—C2—C3—C31	−0.5 (3)	C56—O55—C54—C53	59.1 (3)
C1—C2—C3—C31	178.6 (2)	N52—C53—C54—O55	−59.1 (3)
C2—C3—C4—C5	1.8 (4)	C54—O55—C56—C57	−58.6 (3)
C31—C3—C4—C5	178.4 (2)	C51—N52—C57—C56	−179.0 (2)
C3—C4—C5—C6	6.2 (4)	C53—N52—C57—C56	−57.4 (3)
C3—C4—C5—C51	−174.1 (2)	O55—C56—C57—N52	58.8 (3)
C4—C5—C6—C7	−4.5 (4)	C77—N72—C71—C7	159.88 (19)
C51—C5—C6—C7	175.8 (2)	C73—N72—C71—C7	−77.2 (2)
C5—C6—C7—C1	−6.5 (4)	C6—C7—C71—N72	−6.9 (3)
C5—C6—C7—C71	177.4 (2)	C1—C7—C71—N72	176.48 (19)
O2—C1—C7—C6	−171.5 (2)	C71—N72—C73—C74	174.7 (2)
C2—C1—C7—C6	11.7 (4)	C77—N72—C73—C74	−60.2 (2)
O2—C1—C7—C71	4.8 (3)	C76—O75—C74—C73	−58.6 (3)
C2—C1—C7—C71	−172.0 (2)	N72—C73—C74—O75	60.3 (3)
C33—N32—C31—C3	−145.6 (2)	C74—O75—C76—C77	57.3 (3)
C37—N32—C31—C3	89.6 (2)	C71—N72—C77—C76	−176.41 (19)
C4—C3—C31—N32	9.4 (3)	C73—N72—C77—C76	58.8 (2)
C2—C3—C31—N32	−173.6 (2)	O75—C76—C77—N72	−57.9 (3)

### Hydrogen-bond geometry (Å, °)

**Table:**

*D*—H···*A*	*D*—H	H···*A*	*D*···*A*	*D*—H···*A*
C4—H4···N32	0.95	2.28	2.760 (3)	110
C6—H6···N72	0.95	2.31	2.785 (3)	110
C36—H36A···O01^i^	0.99	2.59	3.497 (3)	153
C53—H53A···O55^ii^	0.99	2.56	3.509 (4)	161

Symmetry codes: (i) *x*−1/2, *y*+1/2, *z*; (ii) −*x*−1/2, −*y*+3/2, −*z*.
